# The Metabolic and Lipidomic Fingerprint of Torin1 Exposure in Mouse Embryonic Fibroblasts Using Untargeted Metabolomics

**DOI:** 10.3390/metabo14050248

**Published:** 2024-04-25

**Authors:** Rani Robeyns, Angela Sisto, Elias Iturrospe, Katyeny Manuela da Silva, Maria van de Lavoir, Vincent Timmerman, Adrian Covaci, Sigrid Stroobants, Alexander L. N. van Nuijs

**Affiliations:** 1 Toxicological Centre, University of Antwerp, 2610 Antwerp, Belgium; elias.iturrospe@uantwerpen.be (E.I.); adrian.covaci@uantwerpen.be (A.C.); 2 Peripheral Neuropathy Research Group, University of Antwerp, 2610 Antwerp, Belgium; 3 Department of In Vitro Toxicology and Dermato-Cosmetology, Vrije Universiteit Brussel, 1090 Brussels, Belgium; 4 Department of Nuclear Medicine, Antwerp University Hospital, 2650 Antwerp, Belgium

**Keywords:** high-resolution mass spectrometry, mTOR, autophagy, metabolism, lipids

## Abstract

Torin1, a selective kinase inhibitor targeting the mammalian target of rapamycin (mTOR), remains widely used in autophagy research due to its potent autophagy-inducing abilities, regardless of its unspecific properties. Recognizing the impact of mTOR inhibition on metabolism, our objective was to develop a reliable and thorough untargeted metabolomics workflow to study torin1-induced metabolic changes in mouse embryonic fibroblast (MEF) cells. Crucially, our quality assurance and quality control (QA/QC) protocols were designed to increase confidence in the reported findings by reducing the likelihood of false positives, including a validation experiment replicating all experimental steps from sample preparation to data analysis. This study investigated the metabolic fingerprint of torin1 exposure by using liquid chromatography—high resolution mass spectrometry (LC-HRMS)-based untargeted metabolomics platforms. Our workflow identified 67 altered metabolites after torin1 exposure, combining univariate and multivariate statistics and the implementation of a validation experiment. In particular, intracellular ceramides, diglycerides, phosphatidylcholines, phosphatidylethanolamines, glutathione, and 5′-methylthioadenosine were downregulated. Lyso-phosphatidylcholines, lyso-phosphatidylethanolamines, glycerophosphocholine, triglycerides, inosine, and hypoxanthine were upregulated. Further biochemical pathway analyses provided deeper insights into the reported changes. Ultimately, our study provides a valuable workflow that can be implemented for future investigations into the effects of other compounds, including more specific autophagy modulators.

## 1. Introduction

Untargeted metabolomics is the discovery-based study of endogenous small molecules (<1500 Da) and provides comprehensive information about the biochemical profile of a biological system at a given time [1,2,3]. Since changes in the metabolome are highly correlated with phenotypic alterations, metabolomics can provide valuable insights into physiological and pathological processes, as well as identify potential diagnostic biomarkers, potential pharmacotherapeutic targets, and mechanisms of action [4]. Torin1 is a strong and selective ATP-competitive inhibitor of the mammalian target of rapamycin (mTOR) that can effectively block mTORC1 and mTORC2 phosphorylation [5,6]. mTOR is a serine/threonine kinase that functions as the catalytic subunit of mTORC1 and mTORC2 complexes. mTOR complexes consist of multiple subunits, in which mTOR interacts with different binding partners that confer complex-specific functions and coordinately, promote cell growth, proliferation, and survival [7]. This inhibition mimics cellular starvation by regulating different signals, such as the unc-51-like kinase 1 (ULK1) complex, thereby upregulating macro-autophagy [8]. mTOR is a major regulatory protein that is involved in many signaling pathways and controls processes other than autophagy (e.g., protein synthesis and regulation of the actin cytoskeleton) [7,9]. Torin1 is increasingly used in autophagy research due to its potency to induce autophagy, despite its unspecific properties [10]. Torin1 is intended for research purposes and has been used both in vitro and in vivo. Liu et al. discovered that torin1 has a short half-life and low oral bioavailability in mice but displays pharmacodynamic inhibition of both mTORC1 and mTORC2 outputs in the lung and liver [6]. Other studies have explored torin1-induced metabolic changes using a (flux) targeted metabolomics method [11,12]. Additionally, Hosios et al. investigated intracellular lipid rearrangements induced by torin1, with a particular focus on lysosomal dependency [13].

Our goal in conducting this study was to develop a reliable and thorough untargeted metabolomics workflow with the application of torin1 exposure in mouse embryonic fibroblast (MEF) cells. Crucially, our quality assurance and quality control (QA/QC) protocols were designed to increase confidence in the reported findings by reducing the likelihood of false positives. This included the implementation of a validation experiment that replicated the original experiment, starting from sample preparation to data analysis. Additionally, only features selected using a combination of univariate and multivariate statistics from both independent experiments were further annotated, and manual verification was done to ensure the correct fold changes and identification [14,15]. To detect this wide range of metabolites, comprehensive sample preparation combined with highly sensitive and specific analytical methods using complementary techniques is often required [16]. In this study, the effects of torin1 exposure on cell metabolism were investigated using liquid chromatography-high-resolution mass spectrometry (LC-HRMS)-based untargeted metabolomics platforms. These analytical platforms combine hydrophilic interaction liquid chromatography (HILIC)-HRMS and reversed-phase liquid chromatography (RPLC)-HRMS combined with electrospray ionization in positive and negative modes (ESI (+) and ESI (−)) [17,18,19]. Two HILIC methods were used in the metabolomics platform to separate polar metabolites such as organic acids, amino acids, and sugars from the polar sample fraction. The apolar sample fraction was analyzed by the lipidomics platform, which uses RPLC to cover a variety of lipid classes. Overall, the metabolic and lipidomic signatures could be elucidated by comparing the relative difference in signal intensity between biological control samples and biological samples exposed to torin1. This approach helps clarify the molecular mechanisms underlying the complex metabolic process of mTOR inhibition, providing a useful workflow that can be implemented for future investigations into the effects of other compounds, including more specific autophagy modulators.

## 2. Materials and Methods

### 2.1. Materials and Chemicals

MEFs were exposed to torin1 (475991, Merk Millipore, Burlington, MA, USA) and staurosporine (81590, Cayman Chemical, Ann Arbor, MI, USA), which had been pre-dissolved in dimethylsulfoxide (DMSO). MEF cells were seeded in Permanox 1-well Lab-Tek chamber slides from Nunc, Thermo Scientific (Rochester, New York, NY, USA) in Dulbecco’s Modified Eagle Medium (DMEM) (Life Technologies, Carlsbad, CA, USA) supplemented with 10% Fetal Bovine Serum (FBS) and 1% Penicillin-Streptomycin (Life Technologies, Carlsbad, CA, USA). Internal standards (IS) for hippuric acid-(phenyl-^13^C_6_), L-lysine-^13^C_6_−^15^N_2_, leucine-5,5,5-D_3_, D-glucose-^13^C_6_, glyceryl tri-(palmitate-1-^13^C), and cholic acid-2,2,4,4-D_4_ were purchased from Sigma-Aldrich (St. Louis, MI, USA). Caffeine-^13^C_3_ was acquired from Cerilliant Corporation (Round Rock, TX, USA), 18:1-D_7_ lyso-phosphatidylethanolamine (LPE), 18:1-D_7_ lyso-phosphatidylcholine (LPC) from Avanti Polar Lipids (Birmingham, UK), octanoyl-L-carnitine-(N-methyl-D_3_), ceramide [d18:1/18:1-(9Z)-^13^C_18_] and L-phenylalanine-^13^C_9_−^15^N from Cambridge Isotope Laboratories (Andover, MA, USA). Additional materials and chemicals are described in Supplementary Materials Section S1.

### 2.2. Cell Line Generation, Treatment, Viability, and Western Blot Analysis

MEFs were generated according to the protocol by Xu (2005) through timed pregnancy in C57BL/6J mice [20]. A detailed protocol is written in Supplementary Materials Section S2.1. The cells were cultured at 37 °C, 5% CO_2_, and saturated humidity in DMEM supplemented with 10% FBS and 1% Penicillin-Streptomycin. The same clone was used for all exposure experiments. Cell viability was evaluated using the Incucyte^®^ Cytotox Red Dye (Sartorius Cat. No. 4632) to assess xenobiotic-induced cytotoxicity, following established guidelines (see Supplementary Materials Section S2.2) [21]. The concentration of torin1 in this study was selected based on previous experiments in MEFs [22]. On day 1, MEFs were exposed to 1 µM torin1 (i.e., exposure group, *N* = 12), 0.1% DMSO (i.e., vehicle control, *N* = 12), and 100 nM staurosporine (i.e., positive control, *N* = 12) in 250 nM Cytotox Red Dye-containing media. Images were taken every two hours over 20 h, capturing phase contrast and fluorescence at 631 nm in 4 random well positions. Cytotox Red dye-positive cells were quantified using a binary mask. Additionally, cell confluency was tracked for 66 h during the metabolomics exposure using phase-contrast images and the Incucyte^®^ live-cell analysis system. A western blot analysis served as a positive control to determine whether torin1 had any effect in our experimental set-up, specifically targeting the autophagic markers LC3 and SQSTM1 (p62). MEFs were exposed to 1 µM torin1 or 0.1% DMSO for 18 h with and without the addition of 100 nM bafilomycin A1, which inhibits lysosomal degradation and autophagosome-lysosome fusion, during the final 3 h of treatment [23]. Cells were collected in ice-cold PBS and lysed in RIPA buffer (1% Nonidet P-40, 150 mM NaCl, 0.1% SDS, 0.5% deoxycholic acid, 1mM EDTA, 50 mM Tris-HCl, pH 7.5) with complete protease and Phospho-STOP inhibitor mixtures for 20 min on ice and cleared by centrifugation for 15 min at 20,817 rcf. Protein concentration in the cell lysate was quantified by Pierce BCA protein assay kit and equally loaded on a NuPAGE™ 12% Bis-Tris gel. Proteins were blotted on a nitrocellulose membrane, and total protein staining was performed after transfer with No-Stain™ Protein Labeling Reagent kit. Blocking was performed with 5% milk powder in 1X PBS/0.5% Tween-20. Membranes were probed with primary antibodies targeting LC3 and SQSTM1 (p62) proteins serving as markers for autophagy induction, along with the loading control β-actin [24,25]. Membranes were developed using Pierce ECL Plus Western Blotting Substrate and imaged with an Amersham 600 Imager.

### 2.3. Exposure Parameters, Sample Collection, and Sample Preparation

**Figure 1:**
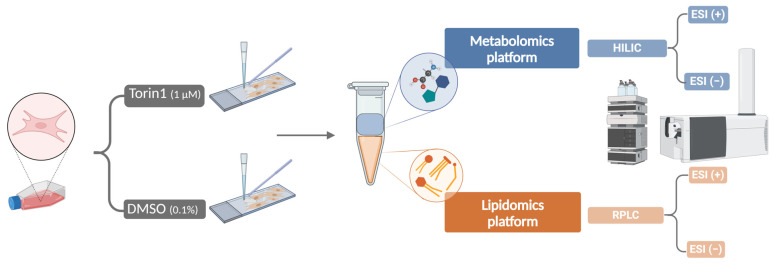
Graphical representation of the MEF cell extract preparation. Cells were exposed to 1 µM torin1 or 0.1% DMSO for 18 h. Liquid-liquid extraction was performed using 3/2/2, MeOH/H_2_O/CHCl_3_ (*v*/*v*/*v*), generating polar and apolar fractions. Each fraction was divided in two for analysis in ESI (+) and ESI (−) modes. ESI, electrospray ionization; HILIC, hydrophilic interaction liquid chromatography; RPLC, reversed phase liquid chromatography.

MEF cells were seeded at a density of 75,000 cells in collagen-coated Permanox 1-well Lab-Tek chamber slides. MEFs were maintained at standard culture conditions to settle and reach confluency. After two days, they were exposed to 1 µM torin1 (*N* = 16) or 0.1% DMSO (negative control, *N* = 16) for 18 h. In addition, four chamber slides containing the culture medium without cells were prepared as extraction blanks. Extraction of metabolites from MEF cells was based on previously described methods [17,26]. A detailed protocol for intracellular MEF cell extract preparation is presented in Supplementary Materials Section S3.1. In short, cells were snap-frozen using liquid nitrogen and scraped from the carrier with 600 µL quenching solution of 80% MeOH and 20% 10 mM CH_3_COONH_4_ (*v*/*v*) in H_2_O at −80 °C. Two chamber slides were combined in one liquid-liquid extraction (LLE) vial with MeOH/H_2_O/CHCl_3_ (3/2/2, *v*/*v*/*v*). Each sample group had eight replicates. A mixture of 12 internal standards, including 6 polar and 6 apolar compounds, was added before extraction. Subsequently, the extraction mixture was vortexed for 60 s, equilibrated for 10 min at 4 °C, centrifuged at 2200× *g* for 7 min at room temperature, and again equilibrated for 10 min at 4 °C. Polar and apolar fractions were transferred and divided into two subfractions before the evaporation step, in order to analyze each subfraction using a different polarity during LC-HRMS acquisitions. Extracts were dried under a N_2_ stream and reconstituted for analysis. Polar and apolar samples were reconstituted using 60 µL of MeCN/H_2_O (65/35, *v*/*v*) and IPA/MeOH (35/65, *v*/*v*), respectively. To separate polar metabolites, two HILIC methods were used on the metabolomics platform. The lipidomics platform, which employs RPLC to analyze a wide range of lipid classes, was used to examine the apolar fraction. Figure 1 provides a graphical representation of the preparation of MEF extracts.

A dilution series was used to evaluate the number of cells needed during sample preparation and the instrumental response of the LC-HRMS system in terms of achieving a balance between sensitivity and detector saturation. A detailed protocol for the optimization of the dilution factor for intracellular MEF cell extracts is included in Supplementary Materials Section S3.2. The appropriate dilution factor was chosen based on the dilution level that enables the instrument to detect most compounds within the linear dynamic range to balance the high and low intensities of metabolites in a sample [27]. In addition, equal volumes from each sample extract (excluding extraction blanks) were combined to generate a separate pooled quality control (QC) sample for each analytical platform and ionization mode. The pooled QC samples were applied to condition the analytical system, acquire MS/MS data, and perform precision measurements over six repeated injections at predefined intervals [28]. To ensure reliable data, a validation experiment was performed, which consisted of an identical replication of the initial experiment from sample preparation to data analysis.

### 2.4. Instrumental Analysis

The data acquisition platforms were previously optimized for both polar and apolar fractions [18,19]. Details on the LC and quadrupole time-of-flight (QToF) parameters can be found in Supplementary Materials Table S1 The polar fraction was analysed on an Agilent 1290 Infinity UPLC system coupled to an Agilent 6530 QToF-HRMS with an Agilent Jet Stream ESI source. In ESI (+), an iHILIC-Fusion column (100 × 2.1 mm, 1.8 μm, zwitterionic, charge modulated amide, silica-based, HILICON AB, Sweden) was used with H_2_O/MeOH (9/1, *v*/*v*) containing 10 mM NH_4_COOH and 0.1% (*v*/*v*) HCOOH as mobile phase A (MPA) and MeCN as mobile phase B (MPB). In ESI (−), an iHILIC-Fusion(P) column (100 × 2.1 mm, 5 μm, zwitterionic, charge-modulated amide, polymer-based, HILICON AB) was used with 2 mM CH_3_COONH_4_ and 2 mM (NH_4_)_2_CO_3_ in H_2_O as MPA and MeCN/MeOH (9/1, *v*/*v*) as MPB.

The analysis of the apolar fraction was performed on an Agilent 1290 Infinity II LC system coupled to an Agilent 6560 drift tube-ion mobility (DTIM)-QtoF-HRMS using Agilent Dual Jet Stream ESI source. In both ESI (+) and ESI (−) modes, an ACQUITY UPLC PREMIER BEH C18 column (150 × 2.1 mm, 1.7 μm, Waters Corporation, Massachusetts, USA) was used with 5 mM CH_3_COONH_4_ in H_2_O/MeCN (7/3, *v*/*v*) as MPA and 5 mM CH_3_COONH_4_ in H_2_O/MeCN/IPA (2/10/88, *v*/*v*/*v*) as MPB. In the ESI (+) mode, 0.1% (*v*/*v*) CH_3_COOH was added to the aqueous fraction of MPA and MPB. During the entire run, a calibrant solution containing purine (*m*/*z* 121.0508 and *m*/*z* 119.0363 in ESI (+) and ESI (−), respectively) and hexakis (1H, 1H, 3H-tetrafluoropropoxy) phosphazine (*m*/*z* 922.0097 and *m*/*z* 966.0007 in ESI (+) and ESI (−), respectively) was constantly infused with an additional isocratic pump to calibrate the mass axis. Samples were randomized, and full scan (MS1) data were acquired in profile mode using 2 GHz extended dynamic range. A pooled QC sample was injected six times at regular intervals [28]. Data-dependent acquisition (DDA) was obtained by injection of the QC pooled sample during system conditioning. DDA with iterative exclusion was also acquired for the lipidomics fractions. During the validation experiment, a target list was used for DDA acquisitions originating from the selected interesting features of the first exposure experiment.

### 2.5. Data Analysis

The raw LC-HRMS data files, stored in the Agilent .d format, were converted to .mzML format using MSConvert [29]. Subsequently, these files were processed in MS-DIAL (v. 4.9) [30]. The specific parameters utilized in MS-DIAL are outlined in Supplementary Materials Table S2 The resulting data matrix was imported into MS-FLO for further deisotoping and duplicate removal [31]. The relative standard deviation (RSD) of the intensity for each feature was plotted for each sample group to assess data quality. Next, drift correction was applied using the cubic spline method in Notame R-package [32]. To guarantee high-quality features, several filtration steps were implemented. Blank subtraction was performed for features with a maximum intensity < 10 times the average extraction blank intensity. Additionally, a feature had to be present in at least 75% of any sample group, and only features with an RSD < 30% on feature intensity in the exposed group were kept. Missing values were imputed by using a random forest (RF), and intensity values were log-transformed [32]. For data normalization, the median intensity of the QC pooled samples was used as a reference for probabilistic quotient normalization (PQN). Subsequently, a Pareto scaling was performed [32,33,34,35,36,37,38,39]. Outlier samples were removed based on the number of features in a sample when compared to other samples from the same group, the principal component analysis (PCA) plots [40], and deviations in the detection of IS. The predefined requirements for IS deviation included a peak height above 5000 counts and without saturation, mass error <10 ppm, retention time (RT) deviation < 0.5 min for HILIC, and <0.2 min for RPLC.

Both univariate and multivariate statistics were used independently to select discriminative features for further annotation. The Shapiro-Wilk test was performed (before data pretreatment) to check for normality, utilizing the intensity values for each feature separately. Depending on the significance (*p* < 0.05) of the Shapiro-Wilk test, a Mann-Whitney U-test or a student-T-test was performed, including a correction for multiple testing [41,42]. Features with *p* < 0.05 and a fold change (FC) > 5 or <0.2 compared to the control group were considered significant. This relatively high threshold for FC was used to keep only the most distinctive metabolites selected by univariate statistics. Our goal in using such strict criteria was to ensure the reproducibility and reliability of our findings. This approach minimizes the likelihood of false positives, improving the validity of our results. Moreover, these stringent selections provide a robust foundation for identifying potential biomarkers that can serve as diagnostic indicators in clinical applications and in vivo experiments, where stringent criteria are often necessary. Additionally, multivariate statistics were performed to take associations into consideration within the broader network. This can help in the biological interpretation during the biochemical pathway analysis of the selected interesting features. Multivariate statistics included a partial least squares-discriminant analysis (PLS-DA) with 7-fold cross-validation and a binary RF classifier [43]. The PLS-DA model was evaluated by a permutation test of the y-variable (*n* = 1000) and by the R^2^ and Q^2^ values of the model, while the RF model was evaluated by the area under the curve (AUC). Interesting features for further annotation were selected based on their variable importance in projection (VIP) value for the PLS-DA model and their mean decrease in accuracy for the RF model. Features selected by the univariate and/or multivariate models were only kept when they were confirmed in a second identical experiment, starting from sample collection to data analysis, to validate and increase the reliability of the results. In addition, boxplots based on the intensity of the features in each group were created for each selected feature and manually evaluated to decrease the number of false positives.

### 2.6. Annotation of Metabolites

A tandem mass spectral library (MS/MS) search was performed to annotate metabolites in both polar and apolar fractions [44]. First, the All Public MS/MS library (v. 15) and modified LipidBlast library were used in MS-DIAL (v. 4.9) [30]. Next, MassBank of North America [45] and the NIST library (v. 17) with MS Search (v. 2.3, National Institute of Standards and Technology, Gaithersburg, MD, USA) [46] were used for MS/MS spectra matching. For the lipid fractions, the rule-based fragmentation tools LipidMatch [47] and LipidHunter [48] were also used. The annotation confidence was improved through manual evaluation of matched MS/MS spectra, utilizing an in-house library (available in MassBankEU 10.5281/zenodo.8308157) for a higher level of confidence if the standard was available, and rule-based fragmentation for manual evaluation of annotated lipids [49,50,51]. All structures were reported according to the annotation confidence system of Schymanski et al. [52] The features that could be annotated with a confidence level (CL) of 3 or higher were reported.

## 3. Results and Discussion

### 3.1. Cell Viability and Western Blot Analysis

**Figure 2:**
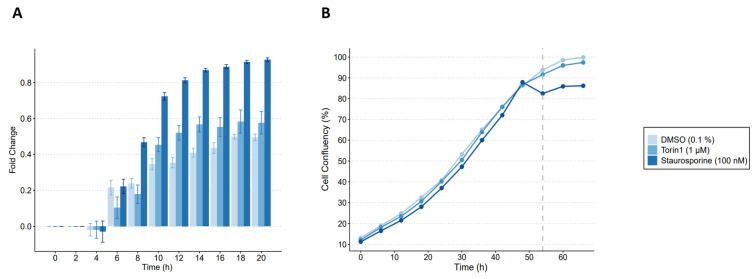
Evaluation of time-dependent cell viability in MEF of torin1 with Incucyte^®^ Cytotox Red Dye: (**A**) normalized fold change in the average red object count per well ± standard error every two hours. MEF were exposed to 0.1% (*v*/*v*) DMSO (i.e., vehicle control, *n* = 12), 100 nM staurosporine (i.e., positive control, *n* = 12), and 1 µM torin1 (i.e., exposure treatment, *n* = 12); (**B**) cell confluency plot over time with exposure of the cells during the last 18 h (dashed grey line).

Torin1 xenobiotic-induced cytotoxicity was evaluated in MEF. Torin1 was compared to vehicle control (0.1% DMSO) and staurosporine, a known cytotoxic compound. The increase in fluorescent emission at 631 nm was used to quantify the reduction in cell viability, which is proportional to the amount of Incucyte^®^ Cytotox Red Dye bound to the DNA of compromised cells. Figure 2A shows the normalized fold changes (FC) in the average red object count per well ± standard error (SE) for all exposure groups at a given time point. Figure 2B shows the percentage of cell confluency during the metabolomics exposure experiment. Exposure to 1 µM torin1 for 18 h showed preserved cell viability with a cell confluency of 97% and a FC increase of 0.2 compared to DMSO. In contrast, exposure to 100 nM staurosporine resulted in decreased cellular viability, as shown by the drop in cell confluency to 85% and the increased FC of 0.8. The western blot analysis (Supplementary Materials Figure S1) served as a positive control and confirmed that torin1 had an effect in our experimental set-up, including the activation of autophagic activity. Together, these results indicate that exposure to 1 µM torin1 for 18 h is appropriate for our metabolic studies, as it has a negligible effect on cell growth and a valid effect on the western blot analysis when compared to the vehicle control.

### 3.2. Data Quality Management

**Figure 3:**
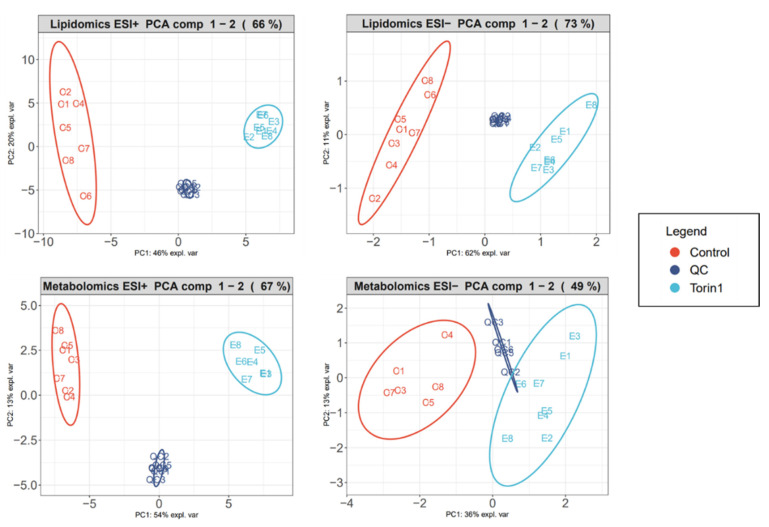
Principal component analysis plots of exposed MEF analyzed in different ionization modes (ESI (+) and ESI (−)) of the first experiment (batch 1). The lipidomics plots refer to the apolar sample fraction, and the metabolomics plots refer to the polar sample fraction. There is a clear distinction between the control group (red) and the sample group exposed to torin1 (light blue), indicating high inter-group variability due to the strong metabolic impact of torin1 exposure. The clustering of quality control (QC) samples (dark blue) indicates limited analytical variation.

After data acquisition with LC-HRMS, the extracted ion chromatograms (EIC) of IS were manually checked to detect outlier samples, and the RSD of the molecular features was calculated per group. For LC-HRMS-based untargeted metabolomics, RSD values ≤ 30% are generally accepted [53,54]. The median RSD (mRSD) of pooled QC samples was calculated to evaluate the analytical precision of the dataset. The QC samples of the polar ESI (−) fraction had the highest mRSD (22%). All other platforms had mRSD < 16% for the QC samples. These values indicate a reliable analytical platform [54]. Supplementary Materials Table S3. provides a summary of all mRSD values for each polarity and treatment group. The higher mRSD for the biological sample group compared to the QC samples can be explained by biological and sample preparation variations. For example, in the apolar ESI (+) fraction, the mRSD increased from 9% to 24%. Similarly, the polar ESI (+) mRSD increased from 13% to 28%. First, a PCA was performed to obtain an indication of the correlations between the variables before using PLS-DA RF [55]. Figure 3 (batch 1) and Supplementary Materials Figure S5 (batch 2) highlights similarities and differences between and within a sample group. PC1 and/or PC2 show a clear separation between the sample groups, indicating high inter-group variability due to the strong metabolic impact of torin1 exposure. The clustering of QC samples demonstrates the repeatability of the instrument and implies that the observed separation between groups is an inherent characteristic of the sample itself [56]. Less clustering of the QC samples in the metabolomics ESI (−) method suggests that this platform was less stable and introduced more analytical variation compared to the other platforms.

Next, PLS-DA and RF models were built to select interesting features for further annotation. As PLS-DA is prone to overfitting, the chemometric model was validated by 7-fold cross-validation and permutation of the y-variable (n permutations = 1000). The PLS-DA model was evaluated by R^2^, Q^2^, R^2^PERM, and Q^2^PERM parameters, and the RF model by AUC (Table 1). The evaluation parameters R^2^ (≥0.977) and Q^2^ (≥0.923) showed high values and low values for R^2^PERM (≤0.002) and Q^2^PERM (≤0.002). The AUC for the RF model evaluation was equal to one for all platforms. Based on the generated evaluation parameters, all models were considered reliable for selecting interesting features.

### 3.3. Metabolic Fingerprint of Torin1 Exposure in MEF Cells

The features selected by the univariate and/or multivariate statistics were kept for annotation after they were validated through a second complete independent replicate of the experiment, starting from the sample collection to the analysis. Supplementary Materials Table S4 lists the annotated metabolites along with their observed retention time, *m*/*z*, ionization species, annotation level, and the software used for MS/MS matching. Following statistical analysis, a total of 84 features could be annotated. These features included multiple ionization species of single metabolites, resulting in a total of 67 annotated metabolites. Of these 67 metabolites, 13% were polar metabolites, and 87% were lipids. Various databases and software tools were used to increase the annotation coverage. For polar metabolites, an in-house library, the All Public MS/MS library (v. 15) in MS-DIAL, NIST (v. 17), and MoNa were used. The in-house library could confirm four of the five polar metabolites with a confidence level of 1. Glycerophosphocholine (GPC) could be annotated with a confidence level of 2a by NIST and MoNa. To annotate the apolar fraction, the All Public MS/MS library (v. 15) and the modified LipidBlast library in MS-DIAL, LipidMatch, and LipidHunter were used. MS-DIAL covered 76% of the annotated lipids but resulted in more false positives and more CL 3-annotated lipids (27%) than LipidMatch. The latter covered 53% of all annotated lipids, with 97% of these having CL 2a. LipidHunter had the least amount of lipids covered, with only 20% coverage.

**Figure 4:**
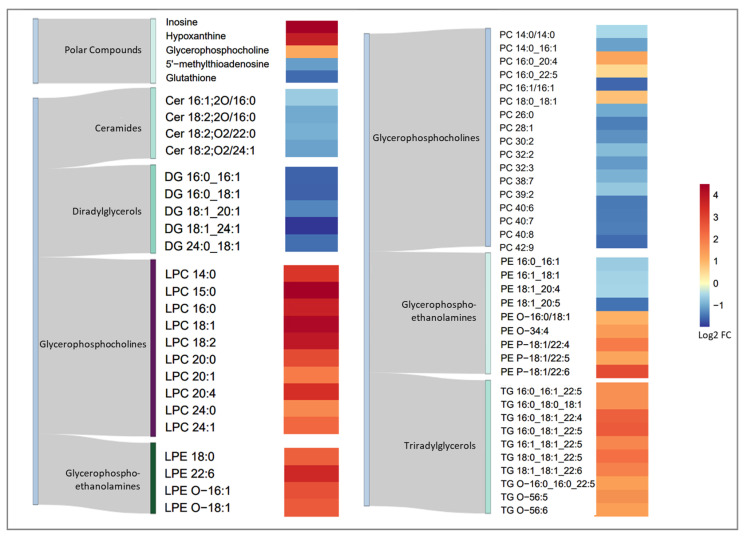
Heatmap of the annotated metabolites with their normalized fold changes to show the intracellular metabolic effect of torin1 exposure in MEF cells. A Sankey diagram was combined to assign classes according to the LIPID MAPS classification system [57]. FC: fold change, Cer: ceramide, DG: diglyceride, LPC: lysophosphatidylcholine, LPE: lysophosphatidylethanolamine, LPE-O: alkyl ether lyso-phosphatidylethanolamine, PC: phosphatidylcholine, PE: phosphatidylethanolamine, PE-O: alkyl ether phosphatidylethanolamine, PE-P: alkenyl ether phosphatidylethanolamine, TG: triglyceride, TG-O: alkyl ether triglyceride.

A heatmap of the normalized fold changes and a Sankey diagram to categorize each metabolite visualizes the effects of torin1 exposure on each individual annotated metabolite (Figure 4). In the polar fraction, inosine and hypoxanthine were strongly upregulated in the exposed group. GPC was also upregulated, but 5′-methylthioadenosine (MTA) and glutathione (GSH) were downregulated as a result of torin1 exposure. In the apolar fraction, ceramides (Cer) with d18:2 and d16:1 backbones were downregulated. All diacylglycerols (DG) were downregulated, with DG 18:1_24:1 having the largest negative fold change. Lyso-phosphatidylcholines (LPC), lyso-phosphatidylethanolamine (LPE), and ether-LPEs were all upregulated, with LPC having the largest fold change of any of the lipids. The majority of intracellular phosphatidylcholines (PC) were downregulated, except for PC 16:0_20:4, PC 16:0_22:5, and PC 18:0_18:1, which showed a slight upregulation. Phosphatidylethanolamines (PEs) were reduced during exposure, but ether-PEs, all triglycerides (TGs), and ether-TGs were more abundant in the treated MEFs.

### 3.4. Metabolic Pathway Analysis

**Figure 5:**
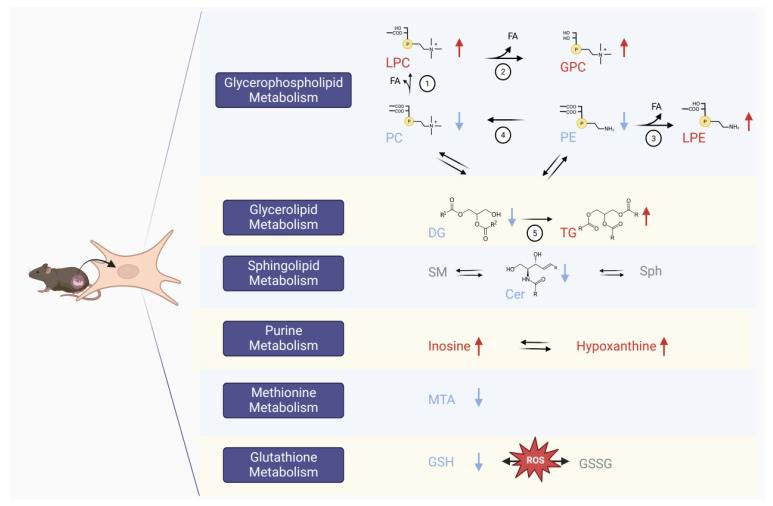
Metabolic pathways that are involved during torin1 exposure in MEF. Blue names and arrows indicate downregulation; red names and arrows indicate upregulation. The numbers that indicate different reactions are discussed in detail in Section 3.4. Cer: ceramide, DG: diglyceride, FA: fatty acid side chain, GPC: glycerophosphocholine, GSH: glutathione, GSSG: glutathione disulfide, LPC: lysophosphatidylcholine, LPE: lysophosphatidylethanolamine, MTA: 5′-methylthioadenosine, PC: phosphatidylcholine, PE: phosphatidylethanolamine, SM: sphingomyelin, Sph: sphingosine, TG: triglyceride.

To gain better insights into the functional roles of metabolites in cellular processes, a metabolic pathway analysis was performed. First, a more general approach was used to identify and evaluate the relationships between the annotated altered lipid species during torin1 exposure. Supplementary Materials Figure S7 shows the network built using the lipid network explorer (LINEX^2^), which visualizes fatty acid and lipid class metabolisms [58]. Next, KEGG was used to explore key disrupted metabolic pathways, as shown in Figure 5, showcasing interactions between these pathways [9].

The metabolism of glycerophospholipids was disturbed after exposure to torin1. The majority of intracellular PCs showed a downregulation, except for PC 16:0_20:4, PC 16:0_22:5, and PC 18:0_18:1, which showed a slight upregulation. Upregulation was observed for LPCs and GPC. These findings suggest that the catabolism of PCs was enhanced during torin1 exposure due to the hydrolysis of the fatty acid side chains from downregulated PC species by phospholipase A1/A2 (PLA1/2) to form the upregulated LPC species (Figure 5 reaction 1). Further hydrolysis of LPCs by lysophospholipase I (LYPLA1) produced a second fatty acid and a glycerophosphodiester headgroup, resulting in the upregulation of the synthesis of GPC (Figure 5, reaction 2) [9].

An alternative explanation is that the decrease in PCs may be the result of their consumption in the synthesis of TGs, while the increase in LPC levels may be a result of increased cellular uptake from the extracellular part to compensate for the reduced levels of PCs. The catabolism of PE was also enhanced during torin1 exposure. The upregulated LPE species can be produced by the hydrolysis of the fatty acid side chains from the decreased PE species, catalyzed by PLA2 (Figure 5, reaction 3). Additionally, a reduction in PE levels may contribute to the downregulation of PC, as phosphatidylethanolamine N-methyltransferase (PEMT) converts PE into PC (Figure 5, reaction 4) [9]. The decreased availability of PE could result in a decrease in the formation of PCs.

The metabolism of glycerolipids was also disturbed by the exposure to torin1. All annotated diacylglycerols were downregulated, with DG 18:1_24:1 showing the highest negative normalized fold change. Triglycerides and ether-triglycerides were all upregulated. In Figure 5, reaction 5 shows the final step of TG biosynthesis from DG and a fatty acyl-CoA, which is catalyzed by diacylglycerol acyltransferase 1 and 2 (DGAT1 and DGAT2) [9]. The increased biosynthesis of TG out of DG can explain these results. Through transferase reactions, the glycerolipid metabolism is also linked to the glycerophospholipid metabolism pathways [9]. A decreased conversion into PC and PE can result from a decreased availability of DG, and vice versa. Another hypothesis is that the fatty acids that become available from increased phospholipid catabolism are stored for energy as TGs, increasing intracellular TG levels [13].

Ether lipids are a type of glycerophospho- and glycerolipids formed in the peroxisome. They are characterized by the presence of an ether bond in which an alkyl or alkenyl chain is attached to the sn-1 position of the glycerol backbone, as opposed to an ester bond. Ether lipids play important roles in various biological functions, including regulating the fluidity and dynamics of cell membranes, acting as antioxidants, participating in intracellular signaling, immunomodulation, and impacting cholesterol metabolism [59,60]. The intracellular levels of alkyl ether LPE, PE, TG, and alkenyl PE are all elevated by torin1 exposure. It is known that in certain pathologies there is an association with ether lipids that is either decreased or increased [61]. As the understanding of their role in pathophysiology is still largely unknown, the mechanism or function of the intracellular increase of these lipids remains unclear.

Ceramides are important sphingolipids that act as a central point in the sphingolipid metabolism. Ceramides can undergo anabolic reactions to produce sphingomyelin and different glycosphingolipids or catabolic reactions that generate sphingosine and sphingosine-1-phosphate [9]. Sphingolipids are increasingly being recognized as having regulatory roles at different stages of the autophagic process. On the other hand, autophagy is being recognized for regulating ceramide levels [62]. Elevated ceramide levels have been implicated as a pathogenic factor in many pathophysiological processes, including neurodegeneration [63,64], obesity [65], and diabetes [66]. It has been observed that mTORC2 controls de novo sphingolipid synthesis in yeast by regulating the activity of ceramide synthase through Ypk2 [67]. Guri et al. showed later that hepatic mTORC2 promotes sphingolipid synthesis in a cancer mouse model [68]. In our study, inhibition of mTORC1 and mTORC2 by torin1 leads to a decrease in all annotated ceramides, building further on the implications of sphingolipid metabolism downregulation and its link to mTOR inhibition.

Inosine and hypoxanthine are both metabolites in the purine metabolic pathway that were upregulated in the torin1-exposed MEF cells. Purine nucleoside phosphorylase (PNP), a transferase enzyme, catalyzes the reaction between inosine and hypoxanthine in the purine salvage pathway. Hypoxanthine can be converted to the inosine monophosphate (IMP) nucleotide by hypoxanthine phosphoribosyltransferase (HPRT) or further broken down to uric acid [9]. In a targeted metabolic flux experiment, Ben-Sahra et al. found that mTORC1 promotes de novo synthesis of purine nucleotides [12]. So a possible explanation for our results of increased intracellular levels due to mTOR inhibition is that the cells compensate by increasing the uptake of hypoxanthine and inosine to form purine nucleotides such as IMP. This requires further investigation to confirm our hypothesis.

5′-Methylthioadenosine (MTA) is an intermediate in the methionine salvage pathway that regenerates methionine from S-adenosylmethionine (SAM)-dependent polyamine biosynthesis [69]. So, MTA downregulation can affect methionine and S-adenosylmethionine (SAM) metabolism, which is linked to mTORC1 [70]. Here, we demonstrate that torin1 treatment results in a decrease in MTA, indicating the stimulating role of mTOR in the synthesis of SAM.

Glutathione, a thiol-containing metabolite, is essential for antioxidant defense, intracellular signaling, and redox homeostasis maintenance [71]. There has been growing evidence in the past years that ROS can regulate autophagy in starvation-induced autophagy by altering the redox state, with GSH having a significant impact on the intracellular redox state [72,73]. The decreased levels of GSH after starvation could be linked to the initiation of autophagy [74]. Torin1 exposure in our study caused an intracellular decrease of GSH in MEF cells, altering the redox state. Previous research reported the involvement of mTORC1 in glutathione synthesis, where mTORC1 signaling regulates the abundance of total cellular glutathione in both reduced and oxidized forms [11]. The decreased GSH levels imply that ROS will be elevated by torin1. Exploring the direct measurement of ROS species or lipid peroxidation would be interesting to assess oxidative stress in future investigations to enhance our understanding of the mechanisms involved.

## 4. Conclusions

Torin1 exposure to MEF resulted in a unique metabolic fingerprint of the intracellular metabolome using an untargeted metabolomics approach. Our research includes a comprehensive study that optimized MEF cell extract preparation, assessed cell viability, performed western blot analysis, and implemented various QA/QC steps within the LC-HRMS workflow, including a validation experiment to ensure high-quality data and increased confidence in the reported findings by reducing the likelihood of false positives. Ultimately, 84 features selected after filtering and a combination of univariate and multivariate statistics from both independent experiments were further annotated and manually inspected to ensure correct fold changes and identification. These features included multiple ionization species of one specific metabolite, resulting in a total of 67 annotated unique metabolites. In particular, torin1 exposure induced changes in lipid metabolism, including the glycerophospholipid, glycerolipid, and sphingolipid pathways. Changes were also observed in the purine, methionine, and glutathione metabolisms. Further biochemical pathway analyses provided deeper insights into the reported changes. To expand our understanding of the effects of torin1, we propose the integration of additional methodologies such as using multiple timepoints and heavy isotope tracing experiments. These approaches would provide insights into the kinetic and flux dynamics of torin1 metabolism, enabling a more comprehensive understanding of its impact on the metabolism. Additionally, our results can serve as a valuable starting point for future research, including in vivo investigations exploring the effects of torin1 on lipid profiles, blood glucose levels, insulin levels, and tissue-specific responses. Overall, a reliable and comprehensive untargeted metabolomics workflow with its application to torin1 exposure in MEF cells is presented and can be implemented in future investigations into the effects of other compounds, including more specific autophagy modulators.

## Figures and Tables

**Table 1:** Evaluation parameters of multivariate statistical models. The PLS-DA model was evaluated by R^2^, Q^2^, R^2^_PER_, and Q^2^_PER_, which were calculated after 1000 random permutations. The random forest classification model was evaluated by the area under the curve (AUC).

		Polar ESI (+)	Polar ESI (−)	Apolar ESI (+)	Apolar ESI (−)
Batch 1					
PLS-DA	R^2^	0.999	0.998	0.998	0.989
	Q^2^	0.991	0.933	0.977	0.979
	R^2^_PERM_	0.002	0.001	0.001	0.002
	Q^2^_PERM_	0.002	0.001	0.001	0.002
RF	AUC	1	1	1	1
Batch 2					
PLS-DA	R^2^	0.995	0.992	0.984	0.977
	Q^2^	0.978	0.923	0.976	0.962
	R^2^_PERM_	0.002	0.001	0.001	0.001
	Q^2^_PERM_	0.002	0.001	0.001	0.001
RF	AUC	1	1	1	1

## Data Availability

The data presented in this study are openly available in MassIVE repository with data set identifier MSV000091645.

## Supplementary Materials

The following supporting information can be downloaded at: https://www.mdpi.com/article/10.3390/metabo14050248/s1, Additional experimental details on intracellular MEF cell extract preparation, optimization of the dilution factor for intracellular MEF cell extracts. Figure S1: Western blot analysis, Figure S2: Dilution series, Figure S3: Dilution experiment; total detected features, Figure S4: Dilution experiment; mean intensity plot, Figure S5: PCA plots batch 2, Figure S6: Scatter plot, Figure S7: LINEX lipidomics network title; Table S1: Data acquisition parameters, Table S2: MS-DIAL parameters, Table S3: mRSD (%), Table S4: Annotated metabolites [75,76].
