# Supplementary material for: The Metabolic and Lipidomic Fingerprint of Torin1 Exposure in Mouse Embryonic Fibroblasts Using Untargeted Metabolomics
Source: Metabolites. 2024 Apr 25;14(5):248. doi: 10.3390/metabo14050248 (PMC11123261; doi:10.3390/metabo14050248)
Supplement: Supplementary file 1 [file metabolites-14-00248-s001.zip › metabolites-2973982-supplementary.pdf]

Supplementary information

The Metabolic and Lipidomic Fingerprint of Torin1 Exposure in Mouse Embryonic Fibroblasts Using Untargeted Metabolomics

Rani Robeyns [1,*], Angela Sisto [2], Elias Iturrospe [1,3], Katyeny Manuela da Silva [1], Maria van de Lavoir [1], Vincent Timmerman [2], Adrian Covaci [1], Sigrid Stroobants [4] and Alexander L. N. van Nuijs [1,*]

[1] Toxicological Centre, University of Antwerp, 2610 Antwerp, Belgium; elias.iturrospe@uantwerpen.be (E.I.); adrian.covaci@uantwerpen.be (A.C.)
[2] Peripheral Neuropathy Research Group, University of Antwerp, 2610 Antwerp, Belgium
[3] Department of In Vitro Toxicology and Dermato-Cosmetology, Vrije Universiteit Brussel, 1090 Brussels, Belgium
[4] Department of Nuclear Medicine, Antwerp University Hospital, 2650 Antwerp, Belgium
* Correspondence: rani.robeyns@uantwerpen.be (R.R.); alexander.vannuijs@uantwerpen.be (A.L.N.v.N.)

TABLE OF CONTENTS

S1. MATERIALS AND CHEMICALS

S2. CELL CULTURE

S2.1. Cell line generation and treatment

S2.2. Cell viability

S2.3. Western blot analysis

S3. SAMPLE PREPARATION MEF CELL EXTRACTS

S3.1. Intracellular MEF cell extracts preparation

S3.2. Optimization of the dilution factor for intracellular MEF cell extracts

S3.2.1. Data analysis

S4. DATA ACQUISITION

S4.1. Table data acquisition parameters.

S5. DATA ANALYSIS

S5.1. MS-DIAL parameters used during data processing

S5.2. mRSD of the intensity of LC-MS features for each sample fraction

S5.3. PCA plots of batch1

S6. ANNOTATIONS

S6.1. Scatter plot

S6.2. Lipidomics network analysis

S6.3. Table of annotated metabolites

S1: MATERIALS AND CHEMICALS

The solvents methanol (MeOH), acetonitrile (MeCN), and formic acid (HCOOH, 99%), all ULC/MS – CC/SFC grade were purchased from Biosolve (Valkenswaard, The Netherlands). Ammonium formate (≥99%, $HCOONH_4$), ammonium acetate (CH_3COONH_4), both LC-MS grade, and ammonium carbonate [$(NH_4)_2CO_3$] HPLC grade, Accutase were obtained from Sigma Aldrich (St. Louis, USA). Acetic acid (100%, CH_3COOH) and ammonia solution (25%, NH_4OH), both LC-MS grade, isopropanol for analysis (IPA) (ACS Reagent), chloroform ($CHCl_3$) analytical grade, were purchased from Merck (Darmstadt, Germany). Ultrapure water (H_2O, 18.2 MΩ) was obtained from an Elga Pure Lab apparatus (Tienen, Belgium). L-ascorbic acid (≥99%), butylated hydroxytoluene (≥99%, BHT), and EDTA (99.995%) were purchased from Sigma Aldrich (St. Louis, USA). Eppendorf Safe-Lock tubes, Reacti-Vials, and 0.2 µm nylon centrifugal filters were acquired from Eppendorf, Thermo Scientific, and VWR (Pennsylvania, USA), respectively. Pure, dry nitrogen (AZOTE N28, N2) used for solvent evaporation was obtained from Air Liquide Belge (Liège, Belgium). 384-well plates (PS, small volume) were bought from Greiner Bio-One (Vilvoorde, Belgium).

For the western blot analysis cells were collected in ice-cold PBS and lysed in RIPA buffer (1% Nonidet P-40, 150 mM NaCl, 0.1% SDS, 0.5% deoxycholic acid, 1mM EDTA, 50 mM Tris-HCl, pH 7.5) with complete protease (Roche Applied Science, 4693159001) and Phospho-STOP inhibitor mixtures (Roche Applied Science, 4906837001). Proteins were quantified by Pierce BCA protein assay kit (23225, ThermoFisher Scientific, Rockford, Illinois, USA) and loaded on a NuPAGE™ 12% Bis-Tris gel (12030166, ThermoFisher Scientific, Rockford, Illinois, USA). Proteins were blotted on a nitrocellulose membrane (10600003, Amersham Biosciences Europe GmbH, Freiburg im Breisgau, Germany). No-Stain™ Protein Labeling Reagent kit (A44717, Invitrogen™, ThermoFisher Scientific, Rockford, Illinois, USA) was used for protein staining. 1X PBS/0.5% Tween-20 was purchased from Sigma Aldrich. Membranes were developed using Pierce ECL Plus Western Blotting Substrate (32132, ThermoFisher Scientific, Rockford, Illinois, USA) and imaged with an Amersham 600 Imager.

S2: CELL CULTURE

2.1. Cell line generation and treatment

Mouse Embryonic Fibroblasts (MEF) were generated through timed pregnancy from C57BL/6J mice [20]. The embryos were isolated from the uterus of a pregnant female and carefully separated from the placenta and other membranes. The embryo tissues were minced and dissociated with 0.25% trypsin and were then allowed to adhere in tissue culture flasks of 25 cm² (690175, Greiner). After the removal of tissue debris, the primary MEF culture was established. At passage two, cells were seeded at 150,000 cells/mL in a Corning™ Costar™ 6-well plate and immortalized with the plasmid pSV51 encoding for the SV40 large T antigen. After seven passages from transfection, while the primary MEF proliferation dropped, the positively transfected cells showed a higher proliferation rate and enriched the cell culture. The cells were cultured at 37 ºC, 5% CO_2 and saturated humidity in DMEM supplemented with 10%

FBS and 1% Penicillin-Streptomycin. It is worthy to mention the potential influence of antibiotic treatment, such as penicillin-streptomycin, in cells when further translational experiments are performed to correlate with our findings *in vivo* [75]. The same clone was used for all exposure experiments.

2.2. Cell viability

According to the provider's guidelines, a cell viability assay was performed using the Incucyte® Cytotox Red Dye. The dye is cyanide-based, which is a highly sensitive nucleic acid stain that binds to deoxyribonucleic acid (DNA) when the cell plasma membrane integrity is compromised. Upon binding to DNA, Cytotox Red Dye increases the fluorescent signal at 631 nm. Therefore, red fluorescent cells correspond to cells that lost membrane integrity and can be considered as not viable. On day 0 (t_0), 1000 cells per well were seeded into a 96-well plate starting from a seeding suspension of 10,000 cells/mL. The plate was incubated overnight and monitored using the Incucyte® live-cell analysis system. On day 1, MEFs were exposed to 1 μM torin1 (i.e., exposure group, $N = 12$), 0.1% DMSO (i.e., vehicle control, $N = 12$), or 100 nM staurosporine (i.e., positive control, $N = 12$) in 250 nM Cytotox Red Dye-containing media. Every two hours, phase-contrast images combined with fluorescence images at 631 nm were acquired in 4 random positions per well for 20 h. A binary mask was used to determine the number of Cytotox Red dye-positive cells. In addition, cell confluency was determined with phase-contrast images using the Incucyte® live-cell analysis system during the metabolomics exposure experiment for 66 h in total.

2.3. Western blot analysis

A western blot analysis served as a positive control to determine whether torin1 had any effect in our experimental set-up, specifically targeting the autophagic markers LC3 and SQSTM1 (p62). Figure S1 demonstrates that torin1 exposure in MEF results in an increased LC3-II/LC3-I ratio when compared to the vehicle control DMSO-treated cells, which at this timepoint indicates an increase in autophagosome formation. The accumulation of LC3-II is further increased by the addition of BafA1, which inhibits autophagosome-lysosome fusion and consequently lysosomal degradation. This suggests that torin1 increased autophagic flux. Furthermore, the western blot also shows that torin1 causes increased degradation of SQSTM1, a substrate of autophagy, compared to the control exposed group. The presence of BafA1 prevents this degradation. These results confirm that toin1 had an effect in our experimental set-up, including the activation of autophagic activity for subsequent metabolomics experiments.

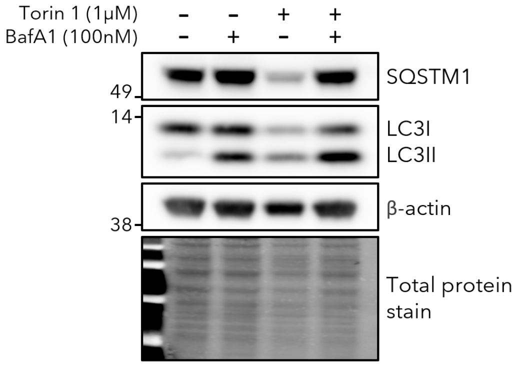

Figure S1 Western blot analysis on autophagy markers LC3-II/LC3-I ratio and SQSTM1 after 18 hours of exposure to torin1 with and without the addition of BafA1, the late autophagy inhibitor, during the final 3 hours. An increased LC3-II/LC3-I ratio during torin1 exposure that further increases with BafA1 and an enhanced SQSTM1 degradation during torin1 exposure indicate enhanced autophagic activity.

S3: SAMPLE PREPARATION MEF CELL EXTRACTS

3.1. Intracellular MEF cell extracts preparation

Immortalized MEF were detached from the cell culture flask using accutase® and seeded in collagen-coated Permanox 1-well Lab-Tek chamber slides, at a concentration of 75 x 10^5 cells per well (day 0). The cells were further incubated for 3 days to reach full confluency at 37 °C and 5% CO_2 in DMEM supplemented with 10% (v/v) Fetal Bovine Serum (heat inactivated at 56 °C for 30 min and steri-filtered) and 1% (m/v) Penicillin-Streptomycin. On day 2, cells were exposed to 1 µM of torin1 (n = 16) and its vehicle DMSO 0.1% (v/v) (i.e., negative control, n = 16) and cultivated for another 18 h. In addition, four extraction blank chamber slides, not containing cells, were obtained using the negative control conditions. After torin1 exposure, approximately 1.2 x 10^6 cells on the chamber slide were washed twice using PBS (37 °C) before snap-freezing with liquid N_2. To quench the metabolome permanently, a solution of 600 µL consisting of 80% (v/v) MeOH and 20% (v/v) of 10 mM NH_4COOCH_3 in H_2O at -80 °C was added to one chamber slide. After 2 min, the cells were scraped and transferred to a vial for liquid-liquid extraction (LLE), which contained 1000 µL of a polar mixture and 840 µL of an apolar mixture (at -20 °C). The polar mixture consisted of 1 mM $(NH_4)_2EDTA$ and 0.5 mM ascorbic acid in 5 mM NH_4COOCH_3 with 0.1% (v/v) $HCOOCH_3$ (pH 4.2). The apolar mixture consisted of 1 mM butylated hydroxytoluene (BHT) in $CHCl_3$.

Internal standards were divided into two mixtures, the first containing 14 µg/mL hippuric acid-(phenyl-$^{13}C_6$), L-lysine-$^{13}C_6$-$^{15}N_2$, leucine-5,5,5-D_3, D-glucose-$^{13}C_6$, caffeine-$^{13}C_3$ and L-phenylalanine-$^{13}C_9$-^{15}N in H_2O/MeOH (1/1, v/v). The second containing 22 µg/mL of cholic acid-2,2,4,4-D_4, glyceryl tri(palmitate-1-^{13}C), 18:1-D_7 lyso-PE, 18:1-D_7 lyso-PC, octanoyl-L-carnitine-(N-methyl-D_3) and ceramide (d18:1/18:1(9Z)-$^{13}C_{18}$) in $CHCl_3$. IS mixtures were added to the polar and apolar mixture to gain a final concentration of 2 µg/mL after reconstitution. Two chamber slides were combined into a single LLE vial, resulting in 8 sample replicates per group and 2 extraction blanks. Subsequently, the

extraction mixture was vortexed for 60 s, equilibrated for 10 min at 4 °C, centrifuged at 2,200 g for 7 min at room temperature, and again equilibrated for 10 min at 4 °C. A volume of 1800 µL of the polar fraction (upper phase) was transferred to an Eppendorf tube, without transferring solid particles from the protein disk. After vortexing for 20 s, 900 µL was transferred to a second Eppendorf tube after which they were evaporated using pure, dry nitrogen at room temperature. 480 µL of the apolar fraction (lower phase) was transferred to a Reacti-Vial. After vortexing for 20 s, 240 µL was transferred to a second Reacti-Vial, after which the liquid was evaporated using pure, dry N_2 at room temperature. Dried extracts were stored at -80 °C and reconstituted right before analysis.

Each fraction (polar and apolar) was divided into two subfractions before the evaporation step, in order to analyze each subfraction using a different polarity during LC-HRMS acquisitions. Polar and apolar samples were reconstituted using 60 µL of MeCN/H_2O (65/35, v/v) and IPA/MeOH (35/65, v/v), respectively. After vortexing for 60 s, samples were filtered over 0.2 µm nylon centrifugal filters and centrifugated at 14,000 g for 2 min at room temperature. Ten µL of each sample (with exception of the extraction blanks) was transferred to a separate LC-vial to create a QC pooled sample. Another 20 µL of each sample was transferred to a Greiner Bio-One 384-well plate (small volume). All samples remained cooled during acquisition in the autosampler (4 °C). The pooled QC samples were applied to condition the analytical system, acquire MSMS data, and perform precision measurements over six repeated injections at a predetermined frequency throughout each run [28]. To ensure reliable data, a second complete independent replicate of the experiment was performed for validation.

3.2. Optimization of the dilution factor for intracellular MEF cell extracts

MEF cell extracts were prepared based on previously described methods [17,26]. A pilot study was conducted to evaluate the number of cells required and to assess our LC-HRMS system to a dilution series of MEF cell extracts. In order to balance the high and low intensities of metabolites in a sample, the appropriate dilution factor was chosen based on the dilution level that enabled the instrument to detect compounds within the linear dynamic range [27]. During the dilution experiment, the same sample preparation method was used as described in section SI–3.1. Fig SI-3.2. gives a graphical overview of the sample preparation and dilution series used. Each chamber slide contained approximately 1.2 x 10^6 cells.

The volume of the quenching solution, polar mix including IS mix, and apolar mix including IS mix were multiplied by factor 3.5 for LLE. A volume of 3150 µL of the polar fraction (upper phase) was transferred to an Eppendorf tube (ESI+). Another volume of 3150 µL of the polar fraction (upper phase) was transferred to an Eppendorf tube (ESI-), without transferring solid particles from the protein disk. 840 µL of the apolar fraction (lower phase) was transferred to a Reacti-Vial (ESI+). Another 840 µL of the apolar fraction (lower phase) was transferred to a Reacti-Vial (ESI-). All extractions were evaporated using pure, dry N_2 at room temperature. Dried extracts were stored at -80 °C and reconstituted directly

before analysis. Polar and apolar samples were reconstituted using 60 µL of MeCN/H_2O (65/35, v/v) and IPA/MeOH (35/65, v/v), respectively. After vortexing for 60 s, serial dilutions were made from the original sample (DIL 0), using the reconstitution solvents as dilution solvent. All samples were filtered over 0.2 µm nylon centrifugal filters and centrifugated at 14,000 g for 2 min at room temperature. 20 µL of each sample from the dilution experiment was transferred to a Greiner Bio-One 384-well plate (small volume). Samples were ordered from low to high concentration for instrumental injection and data acquisition. Each sample was injected *in duplicate*.

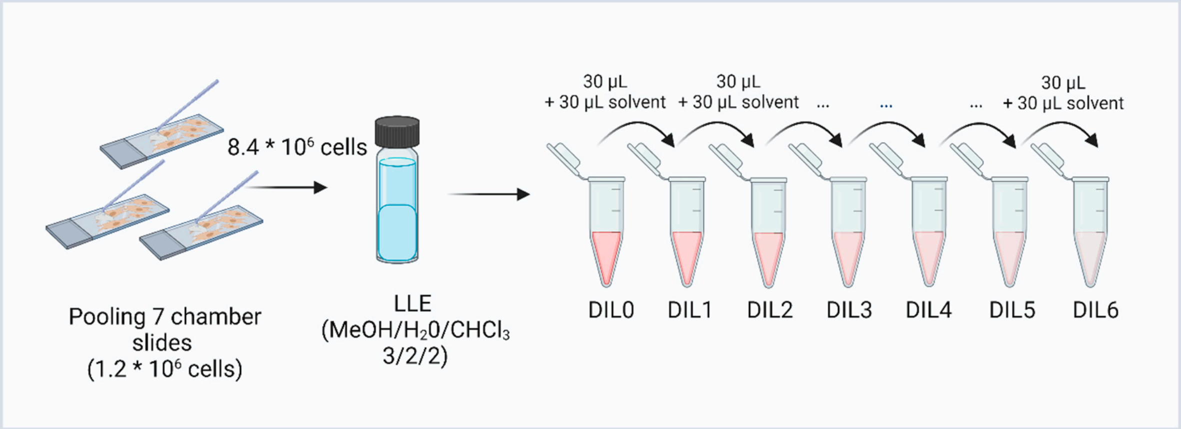

Figure S2 General overview of sample preparation of dilution series used for the optimization of the dilution factor for MEF intracellular extracts. DIL; dilution, LLE; liquid-liquid extraction. Graphical icons were provided by BioRender, license No. 2641-521.

3.2.1. Data analysis

Preprocessing of the acquired data included peak picking, alignment, missing value imputation and solvent blank subtraction. Figure S3 gives a column chart of the number of features detected after these steps according the used analytical platform. Subsequently, the mean intensity values were calculated and log transformed. The Pearson correlation coefficients (r) were calculated for each feature based on the intensity for every combination of four or more consecutive dilution factors. Features with $r > 0.9$ for at least one of the combinations of ≥ 4 consecutive dilution factors were kept. After excluding the features with Pearson correlation coefficients ≤ 0.9, the mean intensity of features was plotted against the dilution factor (Figure S4). For the apolar fraction (lipidomics) in both polarities, there is a smaller increase in mean intensity going from the highest dilution to dilution 3, indicating a high number of features at low intensity. In lipidomics ESI (+), dilution 0 (i.e., the most concentrated sample) showed a very slight bend in the graph. This could indicate a larger number of features closer to the detector saturation level in comparison to dilution 1. For the polar fraction, no indications for detector saturation could be observed in ESI (+). However, in ESI (-), going from dilution 2 to dilution 1 only a small slope was observed, indicating increasing detector saturation. Metabolomics ESI- dilution 0 was confirmed as an outlier, and was not included in the statistics. Based on these results, dilution 2 or dilution 1 would

be suitable concentrations for the apolar fraction, while dilution 2 is preferred for the polar fraction. The sample preparation was adapted accordingly as explained in SI-1.2.

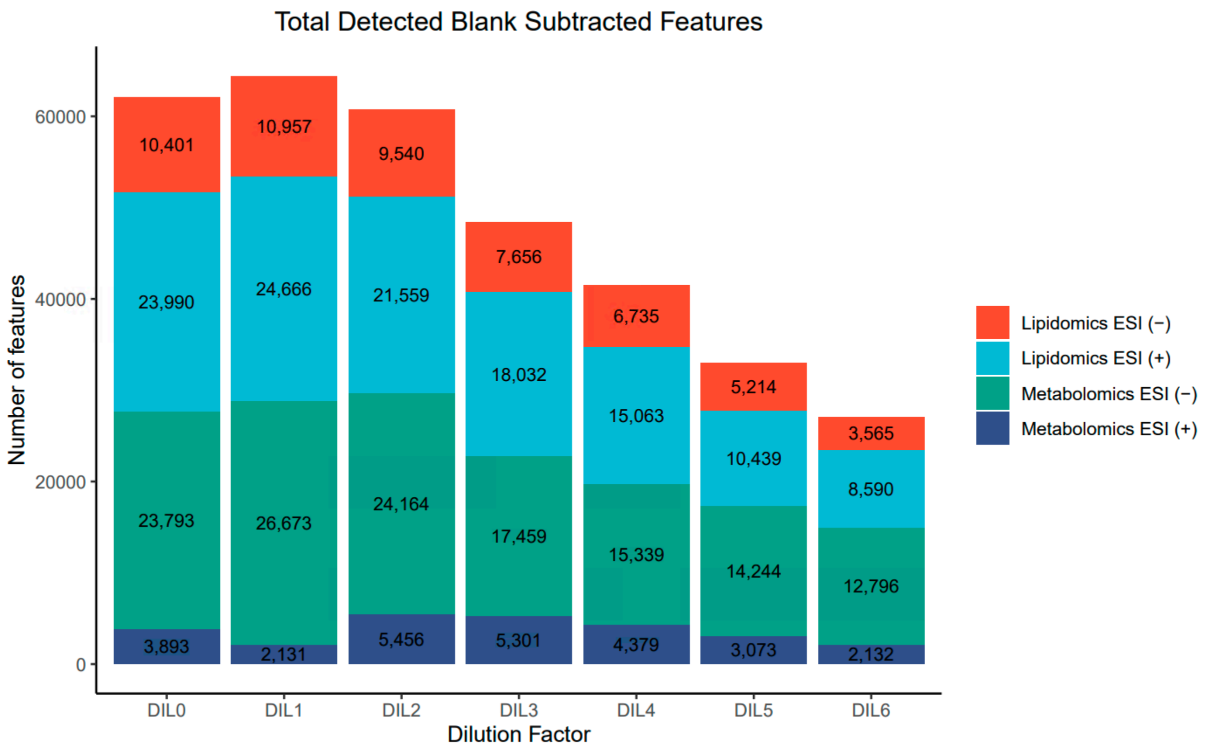

Figure S3 The number of detected features after blank subtraction for each analytical platform. A feature is defined by its *m/z,* retention time and signal intensity or peak area.

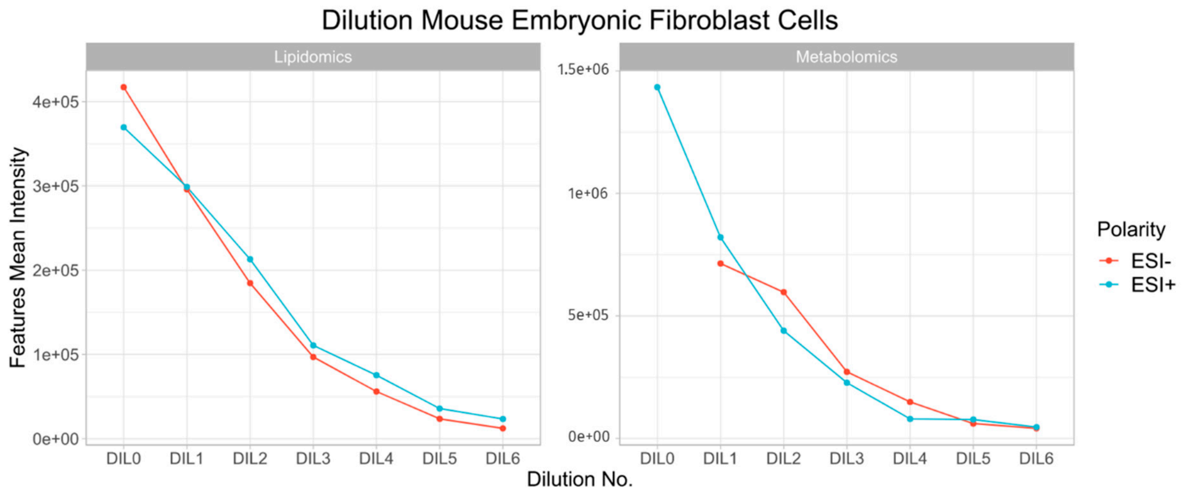

Figure S4 Mean intensity of features plotted against the dilution factor for intracellular lipidomics and metabolomics fractions of MEF.

S4: DATA ACQUISITION

Table S1. Data acquisition parameters.

[a] During the validation experiment a fragmentation target list was used from the selected interesting features of the first exposure experiment.

[b] For the polar fraction, additional MS2 data was acquired using only one collision energy at a time (10, 20 or 40 eV) with maximum 12 precursors per scan cycle. ESI: Electrospray ionization. LC: Liquid chromatography. QToF: Quadrupole-time-of-flight. BEH: Ethylene bridged hybrid. UPLC: Ultra performance liquid chromatography. MeOH: Methanol. MeCN: Acetonitrile. IPA: Isopropanol.

**Sample fraction**	**Polar ESI (+)**	**Polar ESI (-)**	**Apolar ESI (+)**	**Apolar ESI (-)**
**Column**	iHILIC-Fusion	iHILIC-Fusion(P)	ACQUITY UPLC BEH C18	ACQUITY UPLC BEH C18
**Column dimensions**	100 x 2.1 mm, 1.8 µm	100 x 2.1 mm, 5 µm	150 x 2.1 mm, 1.7 µm	150 x 2.1 mm, 1.7 µm
**Mobile phase A**	10 mM NH_4COOH + 0.1% (v/v) HCOOH in H_2O/MeOH (9/1, v/v)	2 mM NH_4COOCH_3 + 2 mM $(NH_4)_2CO_3$ in H_2O	5 mM NH_4COOCH_3 + 0.1% (v/v) $HCOOCH_3$ in H_2O/MeCN (7/3, v/v)	5 mM NH_4COOCH_3 in H_2O/MeCN (7/3, v/v)
**Mobile phase B**	MeCN	MeCN/MeOH (9/1, v/v)	5 mM NH_4COOCH_3 + 0.1% (v/v) $HCOOCH_3$ in H_2O/MeCN/IPA (2/10/88, v/v/v)	5 mM NH_4COOCH_3 in H_2O/MeCN/IPA (2/10/88, v/v/v)
**Flow rate (mL/min)**	0.25	0.20	0.20	0.20
**Gradient**	*Minutes* *%B*<br>*0* 95<br>*4* 95<br>*12.5* 60<br>*20* 60<br>*21* 95<br>*26* 95	*Minutes* *%B*<br>*0* 95<br>*1* 95<br>*10* 20<br>*14* 20<br>*15* 95<br>*20* 95	*Minutes* *%B*<br>*0* 15<br>*2* 15<br>*3* 30<br>*5* 60<br>*8* 60<br>*20* 100<br>*30* 100<br>*35* 15<br>*40* 15	*Minutes* *%B*<br>*0* 15<br>*2* 15<br>*3* 30<br>*5* 60<br>*8* 60<br>*20* 100<br>*30* 100<br>*35* 15<br>*40* 15
**Injection volume (µL)**	3	3	3	2
**Column temperature (°C)**	60	Room temperature (heat exchanger bypass)	60	60

Table S1 Continued; data acquisition parameters

Sample fraction	Polar ESI (+)	Polar ESI (-)	Apolar ESI (+)	Apolar ESI (-)
**Calibrant solution:**				
**Purine (*m/z*)**	121.0508	119.0363	121.0508	119.0363
**Heksakis phosphazine (*m/z*)**	922.0097	966.0007	922.0097	966.0007
**Nozzle voltage (V)**	0	0	500	500
**Capillary voltage (V)**	2000	2000	3500	3750
**Fragmentor voltage (V)**	150	100	200	200
**Drying gas**	Nitrogen	Nitrogen	Nitrogen	Nitrogen
**Sheath gas**	Nitrogen	Nitrogen	Nitrogen	Nitrogen
**Drying gas temperature (°C)**	250	250	325	350
**Sheath gas temperature (°C)**	350	350	325	350
**Drying gas flow (L/min)**	8	10	8	8
**Sheath gas flow (L/min)**	11	10	8	8
**Nebulizer gas pressure (psig)**	45	45	30	30
**MS1 range (*m/z*)**	60-1200	60-1200	100-1500	100-1500
**MS1 acquisition mode**	Profile	Profile	Profile	Profile
**MS1 scan rate (spectra/s)**	2	2	4	4
**MS2 mass range (*m/z*)**	40-1000	40-1000	60-1200	60-1200
**MS2 acquisition mode**	Profile (auto MS/MS)[a]	Profile (auto MS/MS)[a]	Profile (auto MS/MS with iterative exclusion)[a]	Profile (auto MS/MS with iterative exclusion)[a]
**MS2 scan rate (spectra/s)**	6	6	6	6
**Max precursors/scan cycle**	4[b]	4[b]	4	4
**Collision energy (eV)**	10-20-40[b]	10-20-40[b]	10-20-40	10-20-40
**Quad width**	Small (1.3 amu)	Small (1.3 amu)	Small (1.3 amu)	Small (1.3 amu)

S5: DATA ANALYSIS

Table S2 MS-DIAL parameters used during data processing

Sample fraction	Polar ESI (+)	Polar ESI (-)	Apolar ESI (+)	Apolar ESI (-)
**Mass range (Da)**	60-1200	60-1200	100-1500	100-1500
**RT range (min)**	0.5-22	0.5-19.5	0.5-30	0.5-30
**Accurate mass tolerance (MS1) (Da)**	0.01	0.01	0.01	0.01
**Accurate mass tolerance (MS2) (Da)**	0.05	0.05	0.05	0.05
**Maximum charged number**	1	1	1	1
**Smoothing method**	linear weighted moving average	linear weighted moving average	linear weighted moving average	linear weighted moving average
**Scans smoothing level**	3	3	3	3
**Scans minimum peak width**	5	5	5	5
**Mass slice width (Da)**	0.1	0.1	0.1	0.1
**Sigma window value**	0.5	0.5	0.5	0.5
**RT tolerance alignment (min)**	0.2	0.2	0.15	0.15
**MS1 tolerance alignment (Da)**	0.015	0.015	0.01	0.01
**Gap filling**	Yes	Yes	Yes	Yes
**Adduct ion setting**	$[M+H]^+$, $[M+NH_4]^+$, $[M+Na]^+$, $[M-H_2O+H]^+$	$[M-H]^-$, $[M-H_2O-H]^-$, $[M+HCOO]^-$, $[M+CH_3COO]^-$	$[M+H]^+$, $[M+NH_4]^+$, $[M+Na]^+$, $[M-H_2O+H]^+$	$[M-H]^-$, $[M-H_2O-H]^-$, $[M+HCOO]^-$, $[M+CH_3COO]^-$

Table S3 Median relative standard deviation (mRSD) (%) of the intensity of LC-MS features for each sample fraction. mRSD values were calculated after deisotoping and blank subtraction with no gap filling. B1: Batch 1. B2: Batch 2.

	Polar ESI (+) (%)	Polar ESI (-) (%)	Apolar ESI (+) (%)	Apolar ESI (-) (%)
**B1_Control**	19.3	26.6	18.1	24.1
**B1_Torin1**	24.5	28.6	19.1	26.8
**B1_QC**	12.4	20.9	16.0	15.0
**B2_Control**	19.9	35.4	22.1	18.3
**B2_Torin1**	28.2	43.1	24.1	17.3
**B2_QC**	13.1	21.9	9.2	9.0

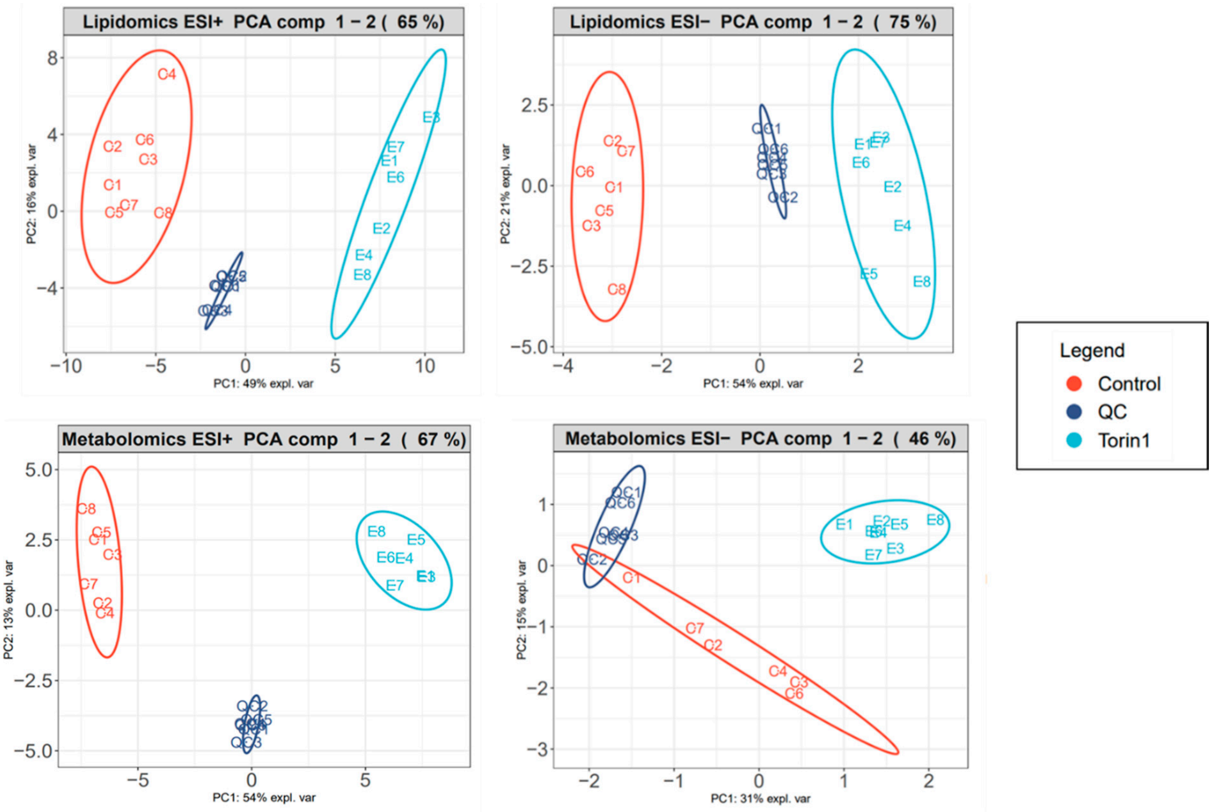

Figure S5 Principal component analysis plots of validation experiment (batch 2) for all sample fractions. The lipidomics plots refer to the apolar sample fraction and the metabolomics plots refer to the polar sample fraction. There is a clear distinction between the control group (red) and the sample group exposed to torin1 (light blue), indicating a high inter-group variability due to a strong metabolic impact of torin1 exposure. The clustering of QC samples (dark blue) indicates a small analytical variation.

S6: ANNOTATIONS

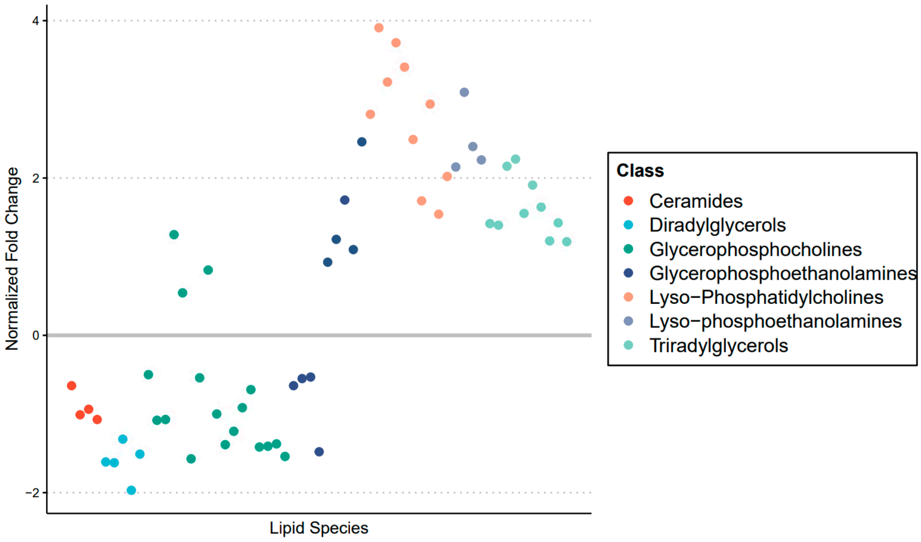

Figure S6 Scatter plot showing the effect of torin1 exposure on the MEF intracellular metabolome. Each dot represents an annotated lipid and is color-coded for a specific lipid class. Most alterations observed in lipid species follow the same effect within a lipid class.

The effect of torin1 exposure on the MEF intracellular lipidome is presented through a scatter plot in Figure S6. The scatter plot shows the normalized fold changes, with each dot representing an annotated metabolite that is color-coded by class. Almost all annotated lipids showed an equal effect within their class. For example, all annotated ceramides (Cer), diradylglycerols (DG), and phosphoethanolamines (PE) showed to be downregulated. Fourteen of the seventeen annotated phosphocholines (PC) were also downregulated, while the remaining three PC were upregulated. All lysophosphatidylcholines (LPC), lysophosphatidylethanolamines (LPE), ether glycerophosphoethanolamines (PE-O), and triradylglycerols (TG) showed an upregulation.

To gain better insights into the functional roles of metabolites in cellular processes, a metabolic pathway analysis was performed. First, a network was built using the lipid network explorer (LINEX²) to identify and evaluate the relationships between the annotated altered lipid species during torin1 exposure (Figure S7). LINEX² is an application that uses publicly available metabolic reaction databases such as Rhea and Reactome and visualizes the fatty acid metabolism and lipid class metabolism [58]. For example, PC 38:5 is connected with a blue line to LPC 16:0, indicating a fatty acid deletion, while its connection with a red line to PE 38:5, indicates a head group modification. PE 38:5 is also connected to PE 38:6 with an orange line, indicating fatty acid modification. Next, the identification of the metabolic pathways that are disturbed during torin1 exposure in the biological system were further explored.

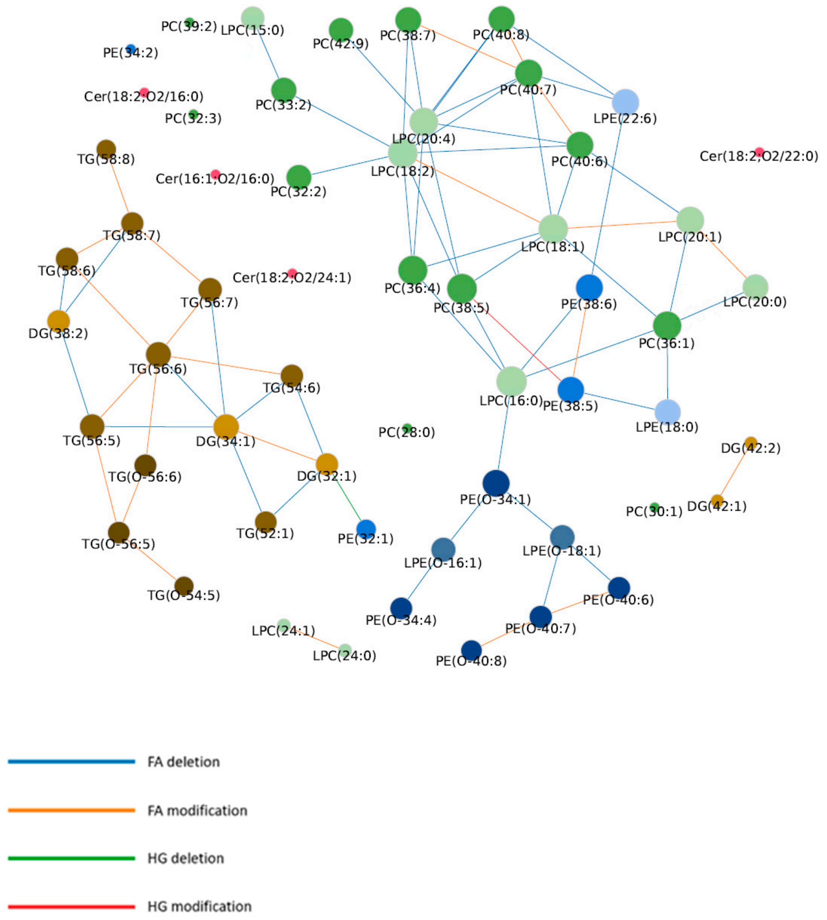

Figure S7 The lipidomics data visualized as a network using lipid network explorer LINEX^2. The lines between lipid species indicate the reaction type. The colour of the dots represents the lipid class and the size depends on the closeness centrality. FA; fatty acid, HG; head group

Table S4 Annotated metabolites of the selected features that showed changes after treatment with torin1. Lipids are classified using the LIPID MAPS classification system [57], while metabolites are classified using the HMDB classification [76]. All structures are reported according to the annotation confidence level system of Schymanski *et al*. (2014) [52] . **Level 3** implies **tentative candidates** when there is insufficient information for one exact structure. **Level 2** implies **a probable structure** and is divided into 2a by matching library or literature data, and 2b by diagnostic evidence. **Level 1** implies a **confirmed structure** by matching with a reference standard.

Bulk Name	Species Name	Subclass	Software Database	Formula	Adduct	*m/z*	RT (min)	Confidence level	Mass error (ppm)
**5'-methylthioadenosine**	5'-methylthioadenosine	5'-deoxy-5'-thionucleosides	In-house library	C11H15N5O3S	[M+H]+	298.0958	4.24	1	-3.4
**Glutathione**	Glutathione	Amino acids, peptides, and analogues	In-house library	C10H17N3O6S	[M+H]+	308.0920	13.41	1	2.9
**Glycerophosphocholine**	Glycero-phosphocholine	Glycerophosphocholines	NIST, MoNa	C8H20NO6P	[M+H]+	258.1100	14.85	2a	-0.4
**Hypoxanthine**	Hypoxanthine	Purines and purine derivatives	In-house library	C5H4N4O	[M+H]+	137.0458	9.06	1	0.0
**Inosine**	Inosine	Purine nucleosides	In-house library	C10H12N4O5	[M+H]+	269.0878	8.98	1	-0.7
**Cer 32:1;2O**	Cer 16:1;2O/16:0	Ceramides	LipidMatch	C32H63NO3	[M-H]-	508.4716	16.62	2a	-3.7
**Cer 34:2;2O**	Cer 18:2;2O/16:0	Ceramides	LipidMatch, MS-DIAL	C34H65NO3	[M-H]-	534.4892	16.87	2a	0.0
**Cer 40:2;O2**	Cer 18:2;O2/22:0	Ceramides	LipidMatch	C40H77NO3	[M-H]-	618.5843	19.12	2a	1.9
			MS-DIAL	C40H77NO3	[M+H-H2O]+	602.5877	19.16	2a	1.2
			LipidMatch	C40H77NO3	[M+H]+	620.5983	19.16	2a	1.1

Table S4 Annotated metabolites (continued)

Bulk Name	Species Name	Subclass	Software Database	Formula	Adduct	*m/z*	RT (min)	Confidence level	Mass error (ppm)
**Cer 42:3;O2**	Cer 18:2;O2/24:1	Ceramides	LipidMatch, MS-DIAL	C42H79NO3	[M-H]-	644.5978	19.14	2a	-1.4
			LipidMatch, MS-DIAL, LipidHunter	C42H79NO3	[M+H]+	646.6135	19.09	2a	0.3
			MS-DIAL	C42H79NO3	[M+H-H2O]+	628.6025	19.09	2a	-0.3
**DG 32:1**	DG 16:0_16:1	Diradylglycerols	LipidMatch, MS-DIAL, LipidHunter	C35H66O5	[M+NH4]+	584.5258	18.21	2a	1.5
			MS-DIAL	C35H66O5	[M+Na]+	589.4812	18.21	2a	1.7
**DG 34:1**	DG 16:0_18:1	Diradylglycerols	LipidMatch, MS-DIAL, LipidHunter	C37H70O5	[M+NH4]+	612.556	18.93	2a	-0.2
			MS-DIAL	C37H70O5	[M+Na]+	617.5120	18.93	2a	0.8
**DG 38:2**	DG 18:1_20:1	Diradylglycerols	LipidMatch, MS-DIAL, LipidHunter	C41H76O5	[M+NH4]+	666.6032	19.54	2a	0.2
**DG 42:1**	DG 24:0_18:1	Diradylglycerols	LipidMatch, MS-DIAL	C45H86O5	[M+NH4]+	724.6804	20.88	2a	-1.4
**DG 42:2**	DG 18:1_24:1	Diradylglycerols	LipidMatch, LipidHunter	C45H84O5	[M+NH4]+	722.6659	20.50	2a	0.3
**LPC 14:0**	LPC 14:0	Glycero-phosphocholines	NIST, MoNa	C22H46NO7P	[M+H]+	468.3092	10.00	2a	1.5
**LPC 15:0**	LPC 15:0	Glycero-phosphocholines	MS-DIAL	C23H48NO7P	[M+H]+	482.3236	8.15	2a	-1.0
**LPC 16:0**	LPC 16:0	Glycero-phosphocholines	MS-DIAL	C24H50NO7P	[M+CH3COO]-	554.3448	8.91	2a	-2.9
**LPC 18:1**	LPC 18:1	Glycero-phosphocholines	MS-DIAL	C26H52NO7P	[M+CH3COO]-	580.3589	8.82	2a	-5.3
			LipidMatch, MS-DIAL	C26H52NO7P	[M+H]+	522.3546	8.87	2a	-1.5

Table S4 Annotated metabolites (continued)

Bulk Name	Species Name	Subclass	Software Database	Formula	Adduct	*m/z*	RT (min)	Confidence level	Mass error (ppm)
**LPC 18:2**	LPC 18:2	Glycero-phosphocholines	LipidMatch	C26H50NO7P	[M+H]+	520.3394	8.16	2a	-0.8
**LPC 20:0**	LPC 20:0	Glycero-phosphocholines	LipidMatch	C28H58NO7P	[M+H]+	552.4014	11.91	2a	-1.8
**LPC 20:1**	LPC 20:1	Glycerophosphocholines	LipidMatch, MS-DIAL	C28H56NO7P	[M+H]+	550.3865	10.63	2a	-0.4
**LPC 20:4**	LPC 20:4	Glycerophosphocholines	LipidMatch, MS-DIAL	C28H50NO7P	[M+H]+	544.3392	8.09	2a	-1.1
**LPC 24:0**	LPC 24:0	Glycerophosphocholines	LipidMatch	C32H66NO7P	[M+H]+	608.4631	15.32	2a	-3.1
			LipidMatch, MS-DIAL	C32H66NO7P	[M+Na]+	630.4468	15.32	2a	-0.2
**LPC 24:1**	LPC 24:1	Glycerophosphocholines	LipidMatch, MS-DIAL	C32H64NO7P	[M+H]+	606.4497	13.90	2a	0.7
			LipidMatch, MS-DIAL	C32H64NO7P	[M+Na]+	628.4316	13.90	2a	0.5
**LPE 18:0**	LPE 18:0	Glycerophospho-ethanolamines	LipidMatch	C23H48NO7P	[M+H]+	482.3242	10.80	2a	0.2
			LipidMatch, MS-DIAL	C23H48NO7P	[M+Na]+	504.3059	10.80	2a	-0.4
**LPE 22:6**	LPE 22:6	Glycerophospho-ethanolamines	LipidMatch, MS-DIAL	C27H44NO7P	[M+H]+	526.2928	8.11	2a	0
**LPE O-16:1**	LPE O-16:1	Glycerophospho-ethanolamines	LipidMatch, MS-DIAL	C21H44NO6P	[M+H]+	438.2976	9.70	2a	-0.7
**LPE O-18:1**	LPE O-18:1	Glycerophospho-ethanolamines	MS-DIAL	C23H48NO6P	[M+H]+	466.3292	11.54	2a	0
**PC 26:0**		Glycerophosphocholines	MS-DIAL	C34H68NO8P	[M+H]+	650.4762	14.26	3	1.1
			MS-DIAL	C34H68NO8P	[M+Na]+	672.4574	14.26	3	-0.1
**PC 28:0**	PC 14:0/14:0	Glycerophosphocholines	MS-DIAL	C36H72NO8P	[M+CH3COO]-	736.5139	15.57	2a	0.7
**PC 28:1**		Glycerophosphocholines	MS-DIAL	C36H70NO8P	[M+Na]+	698.4731	14.47	3	0.0
			MS-DIAL	C36H70NO8P	[M+H]+	676.4902	14.47	3	-1.5
**PC 30:1**	PC 14:0_16:1	Glycerophosphocholines	MS-DIAL	C38H74NO8P	[M+H]+	704.5247	15.64	2a	3.1
			MS-DIAL	C38H74NO8P	[M+Na]+	726.5058	15.64	2a	1.9

Table S4 Annotated metabolites (continued)

Bulk Name	Species Name	Subclass	Software Database	Formula	Adduct	*m/z*	RT (min)	Confidence level	Mass error (ppm)
**PC 30:2**		Glycerophosphocholines	MS-DIAL	C38H72NO8P	[M+H]+	702.5056	15.32	3	-1.7
**PC 32:2**		Glycerophosphocholines	MS-DIAL	C40H76NO8P	[M+H]+	730.5382	15.95	3	0.1
**PC 32:2**	PC 16:1/16:1	Glycerophosphocholines	LipidMatch	C40H76NO8P	[M+Na]+	752.5197	15.95	2a	-0.5
			LipidMatch	C40H76NO8P	[M+CH3COO]-	788.5410	16.10	2a	-4.7
**PC 32:3**		Glycerophosphocholines	MS-DIAL	C40H74NO8P	[M+Na]+	750.5057	15.48	3	1.7
**PC 32:3**		Glycerophosphocholines	MS-DIAL	C40H74NO8P	[M+H]+	728.5226	15.48	3	0.1
**PC 36:1**	PC 18:0_18:1	Glycerophosphocholines	MS-DIAL	C44H86NO8P	[M+CH3COO]-	846.6240	18.38	2a	1.2
**PC 36:4**	PC 16:0_20:4	Glycerophosphocholines	MS-DIAL	C44H80NO8P	[M+CH3COO]-	840.5759	16.85	2a	-0.1
			LipidMatch, MS-DIAL	C44H80NO8P	[M+Na]+	804.5522	16.79	2a	1.0
**PC 38:5**	PC 16:0_22:5	Glycerophosphocholines	MS-DIAL	C46H82NO8P	[M+Na]+	830.5648	16.85	2a	-2.6
**PC 38:7**		Glycerophosphocholines	MS-DIAL	C46H78NO8P	[M+Na]+	826.5310	15.63	3	-5.7
**PC 39:2**		Glycerophosphocholines	MS-DIAL	C47H90NO8P	[M+H]+	828.646	18.65	3	-2.1
**PC 40:6**		Glycerophosphocholines	MS-DIAL	C48H80NO8P	[M+Na]+	856.5831	16.06	3	0.5
**PC 40:7**		Glycerophosphocholines	MS-DIAL	C48H82NO8P	[M+H]+	832.5847	16.66	3	-0.5
			MS-DIAL	C48H82NO8P	[M+Na]+	854.5661	16.66	3	-1.1
**PC 40:8**		Glycerophosphocholines	MS-DIAL	C48H80NO8P	[M+Na]+	852.5497	15.97	3	-2.0
**PC 42:9**		Glycerophosphocholines	MS-DIAL	C50H82NO8P	[M+Na]+	878.5651	16.07	3	-2.2
			MS-DIAL	C50H82NO8P	[M+H]+	856.5856	16.07	3	0.6
**PE 32:1**	PE 16:0_16:1	Glycerophospho-ethanolamines	LipidMatch, MS-DIAL, LipidHunter	C37H72NO8P	[M-H]-	688.4889	16.81	2a	-4.9
**PE 34:2**	PE 16:1_18:1	Glycerophospho-ethanolamines	LipidMatch, MS-DIAL, LipidHunter	C39H74NO8P	[M-H]-	714.5071	17.03	2a	-1.1
**PE 38:5**	PE 18:1_20:4	Glycerophospho-ethanolamines	LipidMatch, LipidHunter	C43H76NO8P	[M-H]-	764.5239	17.33	2a	0.4

Table S4 Annotated metabolites (continued)

Bulk Name	Species Name	Subclass	Software Database	Formula	Adduct	*m/z*	RT (min)	Confidence level	Mass error (ppm)
**PE 38:6**	PE 18:1_20:5	Glycerophospho-ethanolamines	LipidMatch, MS-DIAL, LipidHunter	C43H74NO8P	[M-H]-	762.5074	16.53	2a	-0.7
**PE O-34:1**	PE O-16:0/18:1	Glycerophospho-ethanolamines	LipidMatch	C39H78NO7P	[M-H]-	702.5423	18.14	2a	-2.8
**PE O-34:4**		Glycerophospho-ethanolamines	LipidMatch	C39H72NO7P	[M+H]+	698.5089	15.74	3	-4.3
**PE P-40:5**	PE P-18:1/22:4	Glycerophospho-ethanolamines	MS-DIAL	C45H80NO7P	[M+H]+	778.5741	18.11	2a	-0.5
**PE P-40:6**	PE P-18:1/22:5	Glycerophospho-ethanolamines	LipidMatch, MS-DIAL	C45H78NO7P	[M+H]+	776.5607	17.54	2a	2.3
**PE P-40:7**	PE P-18:1/22:6	Glycerophospho-ethanolamines	MS-DIAL	C45H76NO7P	[M+H]+	774.5439	17.27	2a	0.9
**TG 52:1**	TG 16:0_18:0_18:1	Triradylglycerols	LipidMatch, MS-DIAL, LipidHunter	C55H104O6	[M+NH4]+	878.8170	22.26	2a	-0.1
**TG 54:6**	TG 16:0_16:1_22:5	Triradylglycerols	MS-DIAL, LipidHunter	C57H98O6	[M+NH4]+	896.7696	21.46	2a	-0.7
**TG 56:5**	TG 16:0_18:1_22:4	Triradylglycerols	LipidMatch, MS-DIAL, LipidHunter	C59H104O6	[M+NH4]+	926.8175	21.90	2a	0.4
**TG 56:6**	TG 16:0_18:1_22:5	Triradylglycerols	LipidMatch, MS-DIAL, LipidHunter	C59H102O6	[M+NH4]+	924.7980	21.57	2a	-3.8
**TG 56:7**	TG 16:1_18:1_22:5	Triradylglycerols	LipidMatch	C59H100O6	[M+NH4]+	922.7829	21.29	2a	-3.1
**TG 58:6**	TG 18:0_18:1_22:5	Triradylglycerols	MS-DIAL, LipidHunter	C61H106O6	[M+NH4]+	952.8303	21.86	2a	-2.6

Table S4 Annotated metabolites (continued)

Bulk Name	Species Name	Subclass	Software Database	Formula	Adduct	*m/z*	RT (min)	Confidence level	Mass error (ppm)
**TG 58:7**	TG 18:1_18:1_22:5	Triradylglycerols	LipidMatch, LipidHunter	C61H104O6	[M+NH4]+	950.8179	21.79	2a	0.8
**TG 58:8**	TG 18:1_18:1_22:6	Triradylglycerols	MS-DIAL, LipidHunter	C61H102O6	[M+NH4]+	948.8020	21.50	2a	0.5
**TG O-54:5**	TG O-16:0_16:0_2 2:5	Triradylglycerols	LipidMatch, MS-DIAL	C57H102O5	[M+NH4]+	884.8055	21.99	2a	-1.2
**TG O-56:5**		Triradylglycerols	LipidMatch	C59H106O5	[M+NH4]+	912.8364	22.25	3	-1.6
**TG O-56:6**		Triradylglycerols	LipidMatch	C59H104O5	[M+NH4]+	910.8199	21.97	3	-2.5

REFERENCES

20. Xu, J. Preparation, Culture, and Immortalization of Mouse Embryonic Fibroblasts. *Curr. Protoc. Mol. Biol.* **2005**, *70*, 28.1.1-28.1.8, doi:https://doi.org/10.1002/0471142727.mb2801s70.

75. Ryu, A.H.; Eckalbar, W.L.; Kreimer, A.; Yosef, N.; Ahituv, N. Use Antibiotics in Cell Culture with Caution: Genome-Wide Identification of Antibiotic-Induced Changes in Gene Expression and Regulation. *Sci. Rep.* **2017**, *7*, 7533, doi:10.1038/s41598-017-07757-w.

28. Broadhurst, D.; Goodacre, R.; Reinke, S.N.; Kuligowski, J.; Wilson, I.D.; Lewis, M.R.; Dunn, W.B. Guidelines and Considerations for the Use of System Suitability and Quality Control Samples in Mass Spectrometry Assays Applied in Untargeted Clinical Metabolomic Studies. *Metabolomics* **2018**, *14*, 72, doi:10.1007/s11306-018-1367-3.

17. Iturrospe, E.; da Silva, K.M.; van de Lavoir, M.; Robeyns, R.; Cuykx, M.; Vanhaecke, T.; van Nuijs, A.L.N.; Covaci, A. Mass Spectrometry-Based Untargeted Metabolomics and Lipidomics Platforms to Analyze Cell Culture Extracts. In *Mass Spectrometry for Metabolomics*; González-Domínguez, R., Ed.; Springer US: New York, NY, 2023; pp. 189–206 ISBN 978-1-0716-2699-3.

26. Iturrospe, E.; da Silva, K.M.; Robeyns, R.; van de Lavoir, M.; Boeckmans, J.; Vanhaecke, T.; van Nuijs, A.L.N.; Covaci, A. Metabolic Signature of Ethanol-Induced Hepatotoxicity in HepaRG Cells by Liquid Chromatography–Mass Spectrometry-Based Untargeted Metabolomics. *J. Proteome Res.* **2022**, doi:10.1021/acs.jproteome.2c00029.

27. Wu, Z.E.; Kruger, M.C.; Cooper, G.J.S.; Poppitt, S.D.; Fraser, K. Tissue-Specific Sample Dilution: An Important Parameter to Optimise Prior to Untargeted Lc-Ms Metabolomics. *Metabolites* **2019**, *9*, 1–19, doi:10.3390/metabo9070124.

58. Rose, T.D.; Köhler, N.; Falk, L.; Klischat, L.; Lazareva, O.E.; Pauling, J.K. Lipid Network and Moiety Analysis for Revealing Enzymatic Dysregulation and Mechanistic Alterations from Lipidomics Data. *Brief. Bioinform.* **2023**, bbac572, doi:10.1093/bib/bbac572.

57. Liebisch, G.; Fahy, E.; Aoki, J.; Dennis, E.A.; Durand, T.; Ejsing, C.S.; Fedorova, M.; Feussner, I.; Griffiths, W.J.; Köfeler, H.; et al. Update on LIPID MAPS Classification, Nomenclature, and Shorthand Notation for MS-Derived Lipid Structures. *J. Lipid Res.* **2020**, *61*, 1539–1555, doi:10.1194/jlr.S120001025.

76. Wishart, D.S.; Guo, A.; Oler, E.; Wang, F.; Anjum, A.; Peters, H.; Dizon, R.; Sayeeda, Z.; Tian, S.; Lee, B.L.; et al. HMDB 5.0: The Human Metabolome Database for 2022. *Nucleic Acids Res.* **2022**, *50*, D622–D631, doi:10.1093/nar/gkab1062.

52. Schymanski, E.L.; Jeon, J.; Gulde, R.; Fenner, K.; Ruff, M.; Singer, H.P.; Hollender, J. Identifying Small Molecules via High Resolution Mass Spectrometry: Communicating Confidence. *Environ. Sci. Technol.* **2014**, *48*, 2097–2098, doi:10.1021/es5002105.
